# Suicide HSVtk Gene Delivery by Neurotensin-Polyplex Nanoparticles via the Bloodstream and GCV Treatment Specifically Inhibit the Growth of Human MDA-MB-231 Triple Negative Breast Cancer Tumors Xenografted in Athymic Mice

**DOI:** 10.1371/journal.pone.0097151

**Published:** 2014-05-13

**Authors:** Rosa A. Castillo-Rodríguez, Martha L. Arango-Rodríguez, Lourdes Escobedo, Daniel Hernandez-Baltazar, Anne Gompel, Patricia Forgez, Daniel Martínez-Fong

**Affiliations:** 1 Departamento de Fisiología, Biofísica y Neurociencias, Centro de Investigación y de Estudios Avanzados del Instituto Politécnico Nacional (CINVESTAV), México, D.F., México; 2 Programa de Nanociencias y Nanotecnología, Centro de Investigación y de Estudios Avanzados del Instituto Politécnico Nacional (CINVESTAV), México, D.F., México; 3 Instituto de Ciencias, Facultad de Medicina Clínica Alemana, Universidad del Desarrollo, Santiago, Chile; 4 Unité de Gynécologie, Université Paris Descartes, AP-HP, Port Royal Cochin, Paris, France; 5 Department of Cellular Homeostasis and Cancer, Université Paris Descartes, INSERM UMR-S 1007, Paris, France; Boston University Goldman School of Dental Medicine, United States of America

## Abstract

The human breast adenocarcinoma cell line MDA-MB-231 has the triple-negative breast cancer (TNBC) phenotype, which is an aggressive subtype with no specific treatment. MDA-MB-231 cells express neurotensin receptor type 1 (NTSR1), which makes these cells an attractive target of therapeutic genes that are delivered by the neurotensin (NTS)-polyplex nanocarrier via the bloodstream. We addressed the relevance of this strategy for TNBC treatment using NTS-polyplex nanoparticles harboring the herpes simplex virus thymidine kinase (HSVtk) suicide gene and its complementary prodrug ganciclovir (GCV). The reporter gene encoding green fluorescent protein (GFP) was used as a control. NTS-polyplex successfully transfected both genes in cultured MDA-MB-231 cells. The transfection was demonstrated pharmacologically to be dependent on activation of NTSR1. The expression of HSVtk gene decreased cell viability by 49% (*P*<0.0001) and induced apoptosis in cultured MDA-MB-231 cells after complementary GCV treatment. In the MDA-MB-231 xenograft model, NTS-polyplex nanoparticles carrying either the HSVtk gene or GFP gene were injected into the tumors or via the bloodstream. Both routes of administration allowed the NTS-polyplex nanoparticles to reach and transfect tumorous cells. HSVtk expression and GCV led to apoptosis, as shown by the presence of cleaved caspase-3 and Apostain immunoreactivity, and significantly inhibited the tumor growth (55–60%) (*P*<0.001). At the end of the experiment, the weight of tumors transfected with the HSVtk gene was 55% less than that of control tumors (*P*<0.05). The intravenous transfection did not induce apoptosis in peripheral organs. Our results offer a promising gene therapy for TNBC using the NTS-polyplex nanocarrier.

## Introduction

The targeted gene delivery by synthetic nanoparticles has a potential application for the treatment of aggressive forms of breast cancer, which is the leading cause of cancer deaths among women worldwide [1]. The delivery of therapeutic nanoparticles can be advantageously oriented with the presence of ligands that target an overexpressed or selectively expressed receptor in breast cancer cells. An example is human epidermal growth factor-2 receptor (HER2), which has long been the main target for introducing therapeutic genes in the HER2-enriched breast cancer subtype using polyplexes or lipoplexes that contain a recombinant humanized monoclonal antibody or a single-chain antibody fragment to HER2 as ligands [2]–[4]. However, HER2 cannot be used in the treatment of triple-negative breast cancer (TNBC) because this subtype lacks HER2, along with estrogen and progesterone receptors [5]–[7]. Although TNBC is relatively sensitive to chemotherapy, the lack of a specific treatment presents a poor prognosis to TNBC patients, which present a high risk of relapse within 3 years of diagnosis and an increased mortality rate 5 years after diagnosis [8]–[10]. These issues have prompted the development of more effective therapies against TNBC.

Neurotensin (NTS) receptor type 1 (NTSR1) displays unique structural and functional features to treat different cancers [11]. Transcriptional deregulation in the Wnt/beta-catenin pathway enhances or triggers NTSR1 expression in a great variety of cancer cells, including breast cancer [11]–[16]. Accordingly, NTSR1 expression has been found in 91% of 51 biopsies of patients who were diagnosed with invasive ductal adenocarcinoma, whereas the receptor was absent in normal breast epithelial cells [17]. Recent studies sustain that NTSR1 and NTS play a major role in cancer progression, malignancy, and metastasis because of the development of an autocrine loop [11], [18], [19]. Thus, therapeutic approaches that inhibit expression and function of NTSR1 have proven successful on the human breast adenocarcinoma cell line MDA-MB-231 [17], [20], which has the TNBC phenotype [21], [22]. However, limitations of some approaches that target NTSR1 have not yet been overcome. Although NTSR1 blockage by the systemic administration of its non-peptide antagonist SR 48692 decreases tumor progression, this approach can alter the function of dopaminergic neurons [23], which are known to express high levels of NTSR1. The inhibition of NSTR1 expression by a silencing RNA (siRNA) in MDA-MB-231 cells (NTSR1 (−)) decreases their ability to develop tumors compared with the subtype NTSR1 (+), when xenografted in athymic mice [17]. However, the feasibility of this approach as a treatment for previously established NTSR1 (+) cell tumors remains unknown. Interestingly, NTSR1 internalization, which is overactive in cancer cells [11], has become an efficient pathway to introduce therapeutic genes that are intravenously delivered by the NTS-polyplex nanoparticles as we previously demonstrated in a murine neuroblastoma model [24].

The neurotensin (NTS)-polyplex consists of synthetic nanoparticles that can transfer plasmid DNA (pDNA) encoding any gene of interest into cells that express and internalize the NTSR1, including dopaminergic neurons [25]–[28] and cancer cells [24], [29]. The NTS-polyplex nanoparticles result from the compaction of a pDNA via the electrostatic binding of the Vp1 SV40 karyophilic peptide (KP) and the NTS-carrier, which is a conjugate of poly-L-lysine (PLL), NTS, and the hemagglutinin-derived HA2 fusogenic peptide (FP) [25], [30]. The NTS-carrier promotes gene entry via NTSR1 internalization [25], [27]–[29], [31]. The FP rescues the NTS-polyplex nanoparticles from acidic endosomes, and the KP targets the pDNA to the cell nucleus, which enhances transfection efficiency [25], [26]. The main biophysical properties of the NTS-polyplex nanoparticles that determine the transfection efficiency *in vitro* and *in vivo* have been characterized using radioactive labeling of their peptide components, electrophoretic analysis, and techniques of field emission scanning electron microscopy and transmission electron microscopy [30], [32]. Radioactive peptide conjugation assays have shown that one molecule of NTS and four molecules of FP conjugated with two molecules of PLL in the NTS-carrier produced high efficiency of transgene expression [30]. Electrophoretic analysis of the interactions of NTS-polyplex components revealed that the resulting nanoparticles have neutral charge at optimal molar ratio [30]. At this ratio, the NTS-polyplex nanoparticles fulfill two conditions to cause efficient transfection: an adequate condensation of pDNA into a toroid structure and sufficient concentration of these structures, as shown by transmission electron microscopy studies. These studies together with field emission scanning electron microscopy showed that the NTS-polyplex nanoparticles have an average diameter of 150 nm [30], [32]. A recent study has shown that the intravenous administration of NTS-polyplex nanoparticles does not produce an acute systemic inflammatory response or hepatic cytotoxicity, thus supporting the safety of NTS-polyplex nanoparticles [32]. This property of NTS-polyplex nanoparticles remains important considering the concerns with potential immune reactions to lipoplexes and viral vectors [33], [34], and potential oncogenicity of viral vectors able to integrate the transgene into the host genome [35], [36].

A recent study has demonstrated that the intravenous injection of NTS-polyplex nanoparticles, which are composed of the herpes simplex virus thymidine kinase (HSVtk) gene, and the complementary treatment with ganciclovir (GCV) inhibit the growth of murine neuroblastoma tumors that are allografted in athymic mice [24]. The HSVtk-GCV system is one of the most efficient approaches to cause cell death in rapidly dividing cells [37]. The expressed HSVtk enzyme and the endogenous kinases phosphorylate GCV, which is converted into an active and abnormal triphosphate guanosine analog [38]. Its insertion in elongating DNA by cellular DNA polymerases causes premature chain termination and cell death by apoptosis [38], [39]. The triphosphate GCV produced by the transfected cells may diffuse to neighboring cells to cause apoptosis, a phenomenon known as the “bystander effect” [38], [40], [41]. Even though there are many approaches with other genes that induce apoptosis [42], the HSVtk-GCV system is one of the most frequently used with a proved efficacy in many types of cancer [38].

To date, the therapeutic effectiveness of NTS-polyplex nanoparticles has not yet been explored in human cancer models, including breast cancer. Here, we used the NTS-polyplex nanoparticles for the first time to induce apoptosis in human MDA-MB-231 cells in culture and in xenograft mouse models. Importantly, we demonstrated that the delivery of NTS-polyplex nanoparticles through the bloodstream can inhibit the growth of TNBC in animals without apoptotic effects in peripheral organs. Our results offer a promising therapy for TNBC with the advantage of tumor targeting.

## Materials and Methods

### Plasmids

pEGFP-N1 (4.7 kb) codes for the enhanced green fluorescent protein (GFP) under the control of the cytomegalovirus promoter (Clontech; Mountain View, CA, USA).

pORF-HSVtk (4.373 kb) codes for HSVtk under the hybrid promoter EF-1α/HTLV, which is composed of the Elongation Factor-1 α (EF-1α) promoter and the 5′ untranslated region of the human T-cell leukemia virus (HTLV) (InvivoGen; San Diego, CA, USA).

### Formation of NTS-polyplex Nanoparticles

The detailed procedures for the synthesis of the NTS-carrier and the formation of NTS-polyplex nanoparticles are described elsewhere [30], [31]. Briefly, NTS-polyplex nanoparticles result from the compaction of pEGFP-N1 or pORF-HSVtk plasmids via the electrostatic binding of the Vp1 SV40 karyophilic peptide (KP) and the NTS-carrier, which is a conjugate of poly-L-lysine, NTS, and the hemagglutinin-derived HA2 fusogenic peptide (FP) [25], [30]. We used the criterion of retardation and retention microassays [25], [27] to determine the optimal molar ratio of the NTS-nanocarrier, which was 1 (pDNA): 830 (KP): 24 (NTS-carrier). For the experiments in cell cultures, we used 6 nM of pEGFP-N1 (5.58 µg/300 µL) or pORF-HSVtk (5.19 µg/300 µL) plasmids. For *in vivo* experiments, we injected 300 µL of 30 nM pDNA (i.e., 5X) of those plasmids. Based on the concentration and size of pDNAs, the dose was 1.117 mg/kg of body weight for pEGFP-N1 and 1.039 mg/kg of body weight for pORF-HSVtk. At optimal molar ratio, the concentration of NTS was 126 nM for experiments *in vitro* and 630 nM for experiments in animals, as determined by ^125^I-NTS [30]. Based on the concentration of NTS in 300 µL (injection volume), the dose of the NTS-polyplex in mice was 8.5 nmol/kg of body weight.

### Field Emission Scanning Electron Microscopy

Samples of NTS-polyplex nanoparticles that were prepared in Dulbecco’s Modified Eagle Medium (DMEM) at the optimal molar ratio were placed on a specimen stub (aluminum-iron), which was stuck to double-sided adhesive tape #5085 SPI-AB-Cu. The samples were then dried in a vacuum chamber (Secador™ 1.0 Desiccator cabinet; Bel-Art Products, Wayne, NJ, USA) for 24 h at room temperature. The micrographs were taken in the Advanced Laboratory of Electron Nanoscopy of Centro de Investigación y de Estudios Avanzados del Instituto Politécnico Nacional (CINVESTAV) using a field emission scanning electron microscope (Zeiss Auriga-39-16) with the following parameters: Accelerating voltage, 2 kV; working distance, 3.8 nm; aperture, 7.5 µm; and a secondary electron detector.

### Cell Cultures

MDA-MB-231 cells (ATCC HTB-26) were cultured in RPMI 1640 medium, murine neuroblastoma N1E-115 cells (ATCC CRL-2263) and L-929 mouse fibroblast cells (ATCC CCL-1) were cultured in DMEM, which were supplemented with 10% fetal bovine serum and a mixture of streptomycin-penicillin (100 µg/mL). A clone of NTSR1-silenced MDA-MB-231 cells [17] (SiMDA) was selected with 200 µg/mL hygromicin B (Sigma-Aldrich; St. Louis, MO, USA). The cell lines were incubated at 37°C under an atmosphere of 5% CO_2_ and 99% humidity.

### Delivery of NTS-polyplex Nanoparticles *In* *vitro*

All the internalization and transfection experiments were performed in cells at 80–90% of confluence that were seeded in 24-well plates. Propidium iodide-labeled NTS-polyplex nanoparticles harboring the plasmid pEGFP-N1 were used for the internalization and pharmacological blocking assays in MDA-MB-231 cells following the procedure that was previously described [25], [29], [31]. Briefly, MDA-MB-231 cells were stained with 1 µM calcein green AM (Invitrogen Corporation; Carlsbad, CA, USA) and then incubated with the pharmacological blockers (1 µM NTS or 0.5 µM SR 48692, an NTSR1 non-peptide antagonist, or 0.45 M sucrose) 30 min before and during the 30-min incubation with propidium iodide-labeled NTS-polyplex nanoparticles. Upon the completion of the assay, cells were counterstained with Hoechst 33258 (Sigma-Aldrich; St. Louis, MO, USA) and analyzed using a laser scanning spectral confocal microscope (Leica TCS SPE; Leica Microsystems, Wetzlar, Germany) to detect the fluorescence at Ex-Em wavelengths of 405–461 nm for Hoechst 33258 (blue channel), 488–517 nm for calcein (green channel), and 532–617 nm for propidium iodide (red channel). Ten to twenty consecutive optical sections of 1 µm were taken in a bidimensional plane. The resulting images were projected onto a two-dimensional plane and superimposed on the monitor screen. The positive controls were cells that were exposed to propidium iodide-labeled NTS-polyplex nanoparticles in the absence of pharmacological blockers.

GFP expression in Hoechst 33258-counterstained MDA-MB-231 cells was evaluated 48 h following the exposure to NTS-polyplex harboring the plasmid pEGFP-N1. The fluorescence within the cells was analyzed using a DMIRE2 Leica microscope (Leica Microsystems; Wetzlar, Germany) with filters A for Hoechst 33258 and K3 for GFP. The images were digitized with a Leica DC300F camera (Leica Microsystems, Nussloch, Germany). The negative controls were SiMDA cells, L-929 cells (absence of NTSR1) and untransfected MDA-MB-231 cells. Quantitative analysis of GFP expression assays was achieved using a FACSCalibur flow cytometer (BD Biosciences; San Jose, CA, USA) at 48 h after the transfection of pEGFP-N1 by NTS-polyplex. Cells exposed only to pDNA-KP complex (lacking NTS carrier) were considered as the negative control. Upon completion of expression assays (*n* = 3 independent experiments per condition), cells were trypsinized, suspended in PBS and immediately analyzed by flow cytometry. Populations of 10^4^ cells were excited at the wavelength of 488 nm and the values were plotted according to the dot-plot distribution (Becton Dickinson, Software Cell quest) as was reported previously [25].

The NTS-polyplex harboring the plasmid pORF-HSVtk and GCV (10 µg/mL; Sigma-Aldrich; St. Louis, MO, USA) were used to induce apoptosis in MDA-MB-231 cells.

### Cell Death Assays *In* *vitro*

The proportion of cell death that was induced by HSVtk expression and GCV treatment was determined using three different assays according to the manufacturer’s protocols. 1) The tetrazolium enzymatic conversion to formazan (MTT; Roche Diagnostics Corporation; Indianapolis, IN, USA) was measured by an ELISA reader at 595 nm and at 690 nm [43]. 2) The dye exclusion trypan blue method was used in cells that were harvested with Trypsin-EDTA 0.25%. After the addition of 0.4% trypan blue solution (Sigma-Aldrich; St. Louis, MO, USA) at a 1∶1 ratio (v/v), the unstained cells were counted using a Neubauer chamber. 3) The ApoDETECT annexin V-FITC kit contained propidium iodide (Invitrogen; South San Francisco, CA, USA), which was used to quantify the apoptotic cells. The fluorescence within the cells was quantified in a FACSCalibur flow cytometer (BD Biosciences; San Jose, CA, USA) at Ex-Em wavelengths of 488–522 nm for annexin V FITC or 568–585 nm for propidium iodide. The values were plotted according to the dot-plot distribution (Becton Dickinson, Software Cell quest). All the values were normalized with those values of the negative controls, which were cells without treatment. Staurosporine (1 µM; Sigma-Aldrich Co.; St. Louis, MO, USA) was used to induce apoptosis in MDA-MB-231 cells as a positive of the flow cytometry analysis.

### Reverse Transcription Polymerase Chain Reaction

NTSR1 expression was explored in MDA-MB-231 cells [17], N1E-115 cells (positive control), L-929 and SiMDA cells (negative controls). HSVtk gene expression was explored to show the effectiveness of the transfection *in vitro* and *in vivo*. Total RNA was extracted with guanidinium thiocyanate-phenol-chloroform acid [44] for NTSR1, or TRIzol (Invitrogen Corporation; Carlsbad, CA, USA) for HSVtk, according to the manufacturer’s instructions. Briefly, 1-µg samples of total RNA were used to synthesize cDNA in a reaction mixture of the Super Script III First-Strand Synthesis System (Invitrogen Corporation; Carlsbad, CA, USA) at a final volume of 20 µL at 55°C for 50 min. In the case of HSVtk-transfected cells, the samples were incubated with deoxyribonuclease I (1 U/µL) (Invitrogen; Carlsbad, CA, USA) to rule out the amplification of the plasmid. cDNA amplification was performed using a GeneAmp PCR System 9700 (Applied Biosystems; Grand Island, NY, USA) as follows: 95°C for 10 min, 35 cycles that consisted of denaturation at 94°C for 30 s; annealing for 45 s at 57°C for NTSR1 and 18 S and 58°C for HSVtk; extension at 72°C for 45 s, and a final extension step of 72°C for 10 min. To amplify a 435 bp product of NTSR1, the primers were 5′-CCTTCAAGGCCAAGACCCTC-3′ (forward) and 5′-CAGCCAGCAGACCACAAAGG-3′ (reverse). To amplify a 524 bp product of HSVtk, the primers were 5′-TAATGACAAGCGCCCAGATAA-3′ (forward) and 5′-GGCCCGAAACAGGGTAAATA-3′ (reverse). To amplify a 328 bp product of 18 S (housekeeping gene), the primers were 5′-AGGAATTGACGGAAGGGCAC-3′ (forward) and 5′-GTGCAGCCCCGGACATCTAAG-3′ (reverse). RT-PCR products were fractionated by electrophoresis in a 1.5% agarose gel with 1% TAE buffer at 80 V for 45 min. The gels, which were stained with ethidium bromide, were photographed using a BioDoc-It Imaging System (UVP; Upland, Canada).

### Immunostaining Techniques

The indirect immunofluorescence technique was used to explore NTSR1 protein expression in MDA-MB-231, N1E-115 and L-929 cells. Apoptosis, which was induced by HSVtk expression and GCV treatment, was evaluated in MDA-MB-231 cells using indirect immunofluorescence (caspase-3 activation) [45], [46] and Apostain assays (apoptotic bodies) [45] *in vitro* and *in vivo*. Cultured cells and tissues were processed as previously described [24], [45], [46]. The immunofluorescence staining was performed using the following primary antibodies: a goat polyclonal antibody against NTSR1 (1∶100; Santa Cruz Biotechnology; Santa Cruz, CA, USA) and a rabbit polyclonal antibody against cleaved caspase-3 (1∶400 for *in vitro* and 1∶150 for *in vivo*; Cell Signaling Technology; Beverly, MA, USA). The secondary antibodies were a donkey anti-goat IgG that was conjugated with Alexa Fluor 488 (1∶500; Invitrogen; Eugene, Oregon, USA) and a goat anti-rabbit IgG (H+L) that was conjugated with Texas Red (1∶600 for *in vitro* and 1∶300 for *in vivo*; Vector Laboratories, Burlingame, CA, USA). The samples were counterstained with 1 µM Hoechst 33258. In the case of NTSR1 immunofluorescence, the samples were also counterstained with phalloidin-tetramethylrhodamine B isothiocyanate (TRITC; Sigma-Aldrich; St. Louis, MO, USA). The fluorescence within the samples was analyzed using a laser scanning spectral confocal microscope (Leica TCS SPE; Leica Microsystems; Wetzlar, Germany) at Ex-Em wavelengths of 405–461 nm for Hoechst 33258 (blue channel), 488–519 nm for Alexa 488 (green channel), 532–580 nm for TRITC or 532/620 nm for Texas Red (red channel). Consecutive 1-µm z-series optical sections (10 to 20) were performed and projected in the two-dimensional plane of a monitor.

Condensed chromatin of apoptotic bodies were detected using the mouse monoclonal antibody F7–26 against single-stranded DNA (Apostain; 1∶20; Bender MedSystems; Vienna, Austria) and a rat anti-mouse IgM that was conjugated with peroxidase (1∶300; Invitrogen; South San Francisco, CA, USA) following the procedure that was recently described [45]. The immunostaining was observed using a Leica DM6000 microscope. The images were digitalized with a Leica DFC400 camera (Leica; Nussloch, Germany). Staurosporine (1 µM; Sigma-Aldrich Co.; St. Louis, MO, USA) was used as a positive control of the cleaved caspase-3 immunoassay and Apostain technique.

### Ethics Statement

All procedures were in accordance with the current Mexican legislation, NOM-062-ZOO-1999 and NOM-087-ECOL-1995 (SAGARPA), based on the Guide for the Care and Use of Laboratory Animals, NRC. The CINVESTAV Institutional Animal Care and Use Committee approved our procedures for animal use (protocol #0272-05). All efforts were made to minimize animal suffering.

### Tumor Model in Athymic Mice

Athymic 4-week-old female NuNu-nuBR mice were bred in CINVESTAV facilities. The mice were habituated to the experimental room conditions for 1 week before the study. All the surgical and experimental procedures were performed in mice that were anesthetized with an intraperitoneal (i.p.) injection of an anesthesia mixture (ketamine, 120 mg – xylazine, 24 mg, per kg of body weight) in 0.9% saline solution (Pisa Agropecuaria, Mexico).

Three million MDA-MB-231 cells were subcutaneously injected into the right flank of athymic mice. The cells were previously suspended in serum-free RPMI 1640 medium, which contained growth factor reduced BD matrigel (BD Bioscience; Bedford, MA, USA) at a 1∶1 ratio. The tumors were measured using a digital micrometer (measuring range from 0 to 150 mm with a resolution of 0.01 mm; Truper Inc., Mexico), and their volumes were calculated using the volume formula for an ellipsoid: ½×L×W×H [47].

### Targeted Gene Delivery to MDA-MB-231 Tumors

The internalization and expression assays of NTS-polyplex nanoparticles harboring the plasmid pEGFP-N1 were performed after an injection into tumors at a flow rate of 10 µL/min using a micropump Model MD 1001 (Bionanalytical System Inc.; West Lafayette, IN, USA) or an injection in the bolus via the retro-ophthalmic vein [24]. For the internalization assays, tumors were dissected 6 h after the local injection or 24 h after the intravenous injection of propidium iodide-labeled NTS-polyplex nanoparticles harboring the plasmid pEGFP-N1 [25], [29], [31] as described elsewhere [24]. For GFP expression assays, tumors were dissected on day 3 after the local or intravenous injection [24]. Tumor slices (12 µm) were mounted on glass coverslips and counterstained with fluorescein (FITC)-labeled phalloidin (Invitrogen; Eugene, OR, USA), in the case of internalization assay, or with Hoechst 33258 (Sigma-Aldrich Co.; St. Louis, MO, USA), in the case of expression assays. The fluorescence within the cells was observed with a laser scanning spectral confocal microscope (Leica TCS SPE; Leica Microsystems, Wetzlar, Germany) at Ex-Em of 405–461 nm for Hoechst 33258 (blue channel), 488–516 nm for FITC and GFP (green channel), and 532–617 nm for propidium iodide (red channel).

### Antitumoral Effect of HSVtk Gene Transfection and GCV Treatment

When the tumors reached a volume of 100 mm^3^, NTS-polyplex nanoparticles harboring the plasmid pORF-HSVtk were delivered via local or intravenous injections every 2 days for a 10-day period. GCV (70 mg/kg of body weight, i.p.) was daily injected to activate the HSVtk suicide system [24]. The negative controls were animals that were injected with NTS-polyplex harboring the plasmid pEGFP-N1. The tumor size was measured daily and weighted at the end of the study. At this time, the tumors and tissues (liver, lungs, kidneys, and colon) were processed using indirect immunofluorescence to detect active capase-3 and Apostain as described above.

### Statistical Analysis

All values are provided as the mean ± SEM. After testing for normality with the Snedecor F-analysis, the difference between groups in all the essays was analyzed using a one-way ANOVA, and the difference between the growth rates of tumors was analyzed using a two-way ANOVA test. When the ANOVA showed a significant difference, a comparison between means was analyzed using a *post-hoc* Bonferroni test. All statistical analyses were performed using the GraphPad Prism software (GraphPad Software Inc.; San Diego, CA, USA). *P* values<0.05 were considered significant.

## Results

### Gene Delivery into Cultured MDA-MB-231 Cells via NTSR1 Internalization

**Figure 1:**
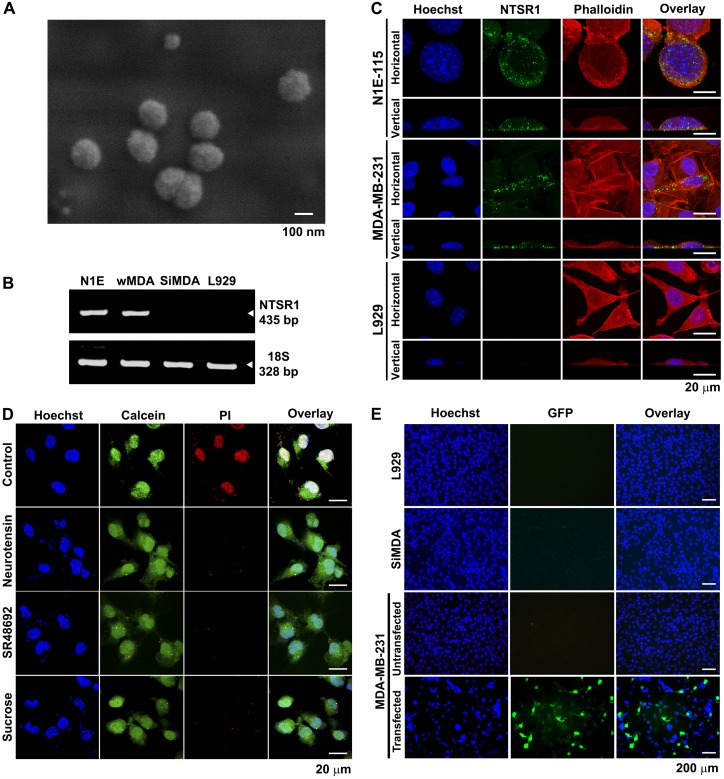
Gene delivery into breast cancer MDA-MB-231 cells via the internalization of NTSR1. (**A**) A representative micrograph of NTS-polyplex nanoparticles that are formed at an optimal molar ratio (1 (plasmid): 24 (NTS-carrier)), which was taken using a field emission scanning electron microscope. (**B**) Representative agarose gel showing the presence of the amplicons for NTSR1 and 18S after an RT-PCR assay. N1E = N1E-115; wMDA = wild type MDA-MB-231 cells; SiMDA = NTSR1-silenced MDA-MB-231 cells. L-929 = mouse fibroblast cell line. (**C**) Representative confocal micrographs of an immunofluorescence assay showing the presence of NTSR1 in MDA-MB-231 cells that were counterstained with TRITC-phalloidin and Hoechst 33258. One-micrometer optical sections in the x–y plane (horizontal) and in the z plane (vertical). (**D**) Representative confocal micrographs of propidium iodide (PI) NTS-polyplex harboring the plasmid pEGFP-N1 in MDA-MB-231 cells in the absence or presence of different pharmacological blockers. The cells were counterstained with Hoechst 33258 and calcein. (**E**) Representative micrographs showing GFP expression in MDA-MB-231 cells after exposure to NTS-polyplex nanoparticles carrying pEGFP-N1 plasmid. The cells were counterstained with Hoechst 33258.

The field emission scanning electron microscopy analysis showed that the NTS-polyplex compacts the plasmid pORF-HSVtk into nanoparticles (∼150 nm) (Figure 1A), as well as the plasmid pEGFP-N1 (Data not shown). Because these NTS-polyplex nanoparticles depend on NTSR1 to enter the target cell [25], [27]–[29], [31], experiments were performed to first demonstrate the presence of functional NTSR1 in MDA-MB-231 cells. RT-PCR and immunofluorescence assays showed the presence of NTSR1 mRNA and protein in MDA-MB-231 cells and in N1E-115 (positive control), but not in SiMDA and L-929 cells (negative control) (Figure 1B and 1C). Accordingly, the confocal microscopy analysis showed propidium iodide spots within the MDA-MB-231 cells 30 min after incubation with propidium iodide-labeled NTS-polyplex nanoparticles (Figure 1D). The fluorescence of propidium iodide was absent in MDA-MB-231 cells that were co-incubated with NTS (1 µM), SR 48692 (0.5 µM), NTSR1 non-peptide antagonist [48], or with 0.45 M sucrose, which is a receptor-mediated endocytosis inhibitor [49]. The results of NTSR1 blockade and receptor-mediated endocytosis inhibition strongly suggest that the transfection is dependent on activation of NTSR1 as was previously reported [25], [26], [28].

Consequently, MDA-MB-231 cells were able to express GFP 48 h after incubation with NTS-polyplex carrying the plasmid pEGFP-N1 (Figure 1E). The percentage of cells expressing GFP was 18.3±0.5% (*n* = 3) as determined by flow cytometry analysis (Figure S1). In contrast, no GFP expression was present in either SiMDA or L-929 cells (negative control), which lacked NTSR1. These results demonstrate the mediation of NTSR1 in the transfection of MDA-MB-231 cells by NTS-polyplex nanoparticles that had NTS as a ligand.

### HSVtk Gene Expression and GCV Treatment Activate Apoptosis in Cultured MDA-MB-231 Cells

**Figure 2:**
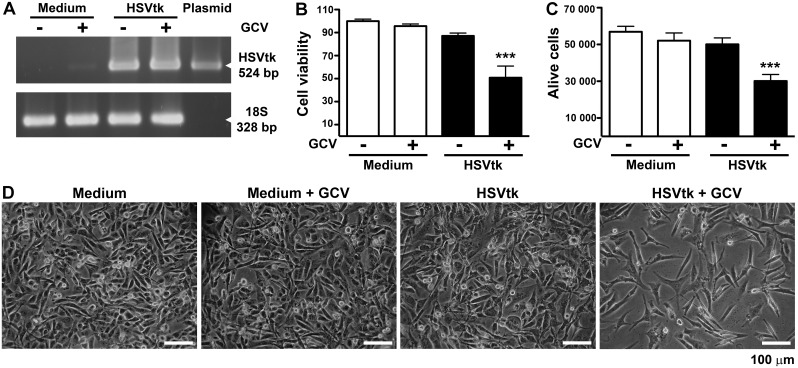
Cell death caused by HSVtk expression and ganciclovir treatment. MDA-MB-231 cells were transfected with NTS-polyplex harboring the plasmid pORF-HSVtk and treated with GCV (10 µg/mL) for 48 h. (**A**) Representative agarose gel showing the presence of the amplicons for HSVtk and 18S after an RT-PCR assay. (**B**) Cell viability using the MTT colorimetric assay (*n* = 5 independent experiments per group). (**C**) Cell counting of live cells using the trypan blue dye exclusion method (*n* = 6 independent experiments per group) (**D**) Representative micrographs in bright field taken 48 h after the treatments. ***P<0.0001 when compared with untransfected and GCV untreated cells. One-way ANOVA and *post-hoc* Bonferroni test.

RT-PCR analysis showed HSVtk gene expression in MDA-MB-231 cells 48 h after exposure to NTS-polyplex nanoparticles harboring the plasmid pORF-HSVtk in the presence or absence of GCV (Figure 2A). However, only cell-death was induced in cells that were treated with GCV. MTT assay showed that the cell viability decreased 49±10% (*P*<0.0001) compared with the controls (Figure 2B). A similar decrease in percentage (47±11%; *P*<0.0001) was determined by the trypan blue method, which excluded dead cells in the counting (Figure 2C). Accordingly, bright field microscopy showed a significant decrease in the cell population after the treatment with NTS-polyplex harboring the plasmid pORF-HSVtk and GCV compared with controls (Figure 2D).

**Figure 3:**
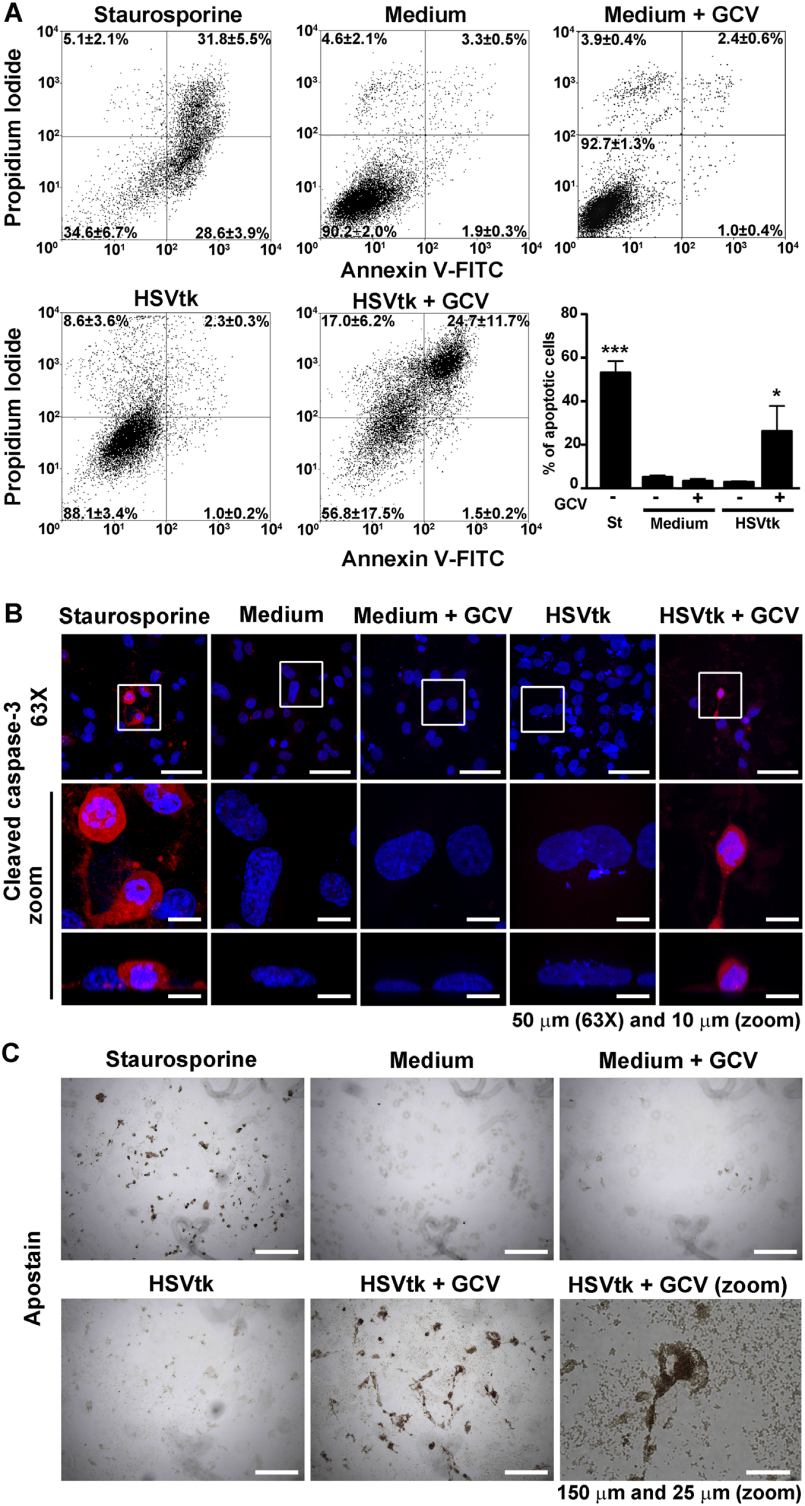
Apoptosis induced by HSVtk expression and ganciclovir treatment. Apoptosis in MDA-MB-231 cells was evaluated 24 h after the exposure to NTS-polyplex harboring the plasmid pORF-HSVtk and GCV (10 µg/mL). (**A**) Representative flow-cytometry dot-plot graphics showing the proportion of cells that were stained with Annexin V-FITC (apoptosis) and propidium iodide (necrosis) whose percentage are expressed as the mean ± SEM in the corresponding quadrant. The insert shows the percentage of apoptotic cells (*n* = 5 per group) which correspond to the sum of the percentage in the inferior right quadrant (showing early apoptosis) plus the percentage in the superior right quadrant (showing late apoptosis). (**B**) Representative confocal micrograph showing the immunofluorescence against cleaved caspase-3 in cells that were counterstained with Hoechst 33258 (*n* = 3 per group). One-micrometer optical sections in the x–y plane (horizontal) and in the z plane (vertical). (**C**) Representative bright field micrographs of condensed chromatin of apoptotic bodies that were stained with Apostain. Staurosporine was used as a positive control of apoptosis. All the assays were performed 24 h after the treatments. *P<0.05, ***P<0.0001 when compared with untransfected and GCV treated cells. One-way ANOVA and *post-hoc* Bonferroni test.

The activation of apoptosis, which was triggered by HSVtk expression and GCV treatment, in MDA-MB-231 cells was supported by coinciding results of the annexin V assay (Figure 3A), cleaved caspase-3 immunostaining (Figure 3B), and the Apostain assay (Figure 3C) that were obtained at 24 h after transfection. The flow cytometry assay showed that 26%±12% of MDA-MB-231 cells were positive to annexin V, which is an apoptosis marker, 24 h after the treatment with NTS-polyplex nanoparticles harboring the plasmid pORF-HSVtk and GCV (Figure 3A). This percentage was significantly different from the negative controls (5±1%; *P*<0.05). The increase in annexin V-positive MDA-MB-231 cells (53±5%; *P*<0.0001), which were treated with staurosporine as a positive control, supports the validity of the assay (Figure 3A). Accordingly, cleaved caspase-3 (Figure 3B) and apoptotic bodies (Figure 3C) were detected only in MDA-MB-231 cells 24 h after the treatment with either NTS-polyplex harboring the plasmid pORF-HSVtk and GCV treatment or staurosporine.

### Targeted Gene Delivery to MDA-MB-231 Cell Tumors in Athymic Mice

**Figure 4:**
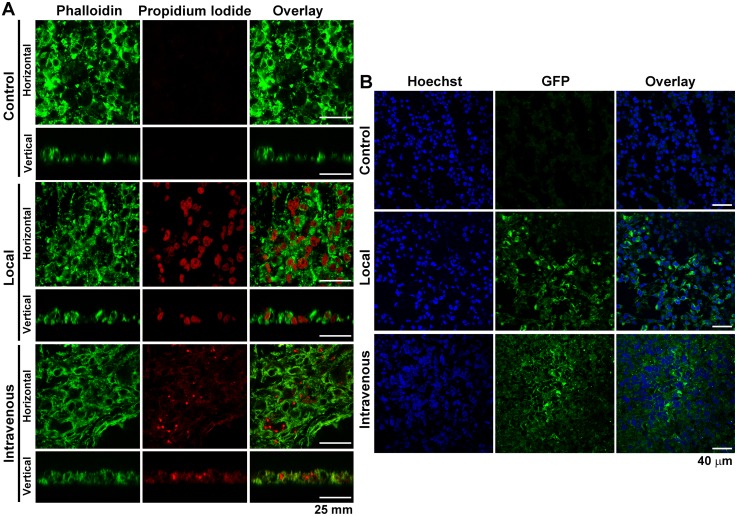
Targeted gene delivery to MDA-MB-231 cell tumors in athymic mice. (**A**) Representative confocal micrographs of cells that were counterstained with FITC-phalloidin taken after a local (6 h) or intravenous (24 h) injection of NTS-polyplex nanoparticles carrying the plasmid pEGFP-N1 labeled with propidium iodide. One-micrometer optical sections in the x–y plane (Horizontal) and in the z plane (vertical). (**B**) Representative micrographs showing the expression of GFP in cells that were counterstained with Hoechst 33258 on day 3 after a local or intravenous injection of NTS-polyplex nanoparticles carrying the plasmid pEGFP-N1.

Consistent with the *in vitro* results, the confocal microscopy analysis showed the presence of propidium iodide fluorescence within the MDA-MB-231 cells xenografted in athymic mice after local (6 h) or intravenous (24 h) injections of propidium iodide-labeled NTS-polyplex nanoparticles harboring the plasmid EGFP-N1 (Figure 4A). Consequently, MDA-MB-231 cells were able to express GFP on day 3 after injection with NTS-polyplex harboring the plasmid pEGFP-N1 (Figure 4B).

### Antitumoral Therapy Mediated by HSVtk Gene Expression and GCV Treatment

**Figure 5:**
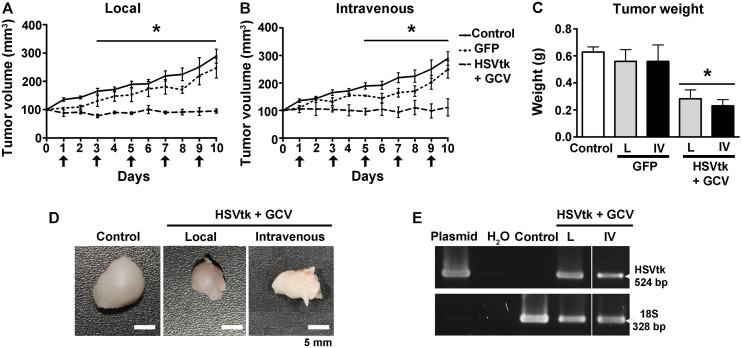
Inhibition of tumor growth by HSVtk expression and ganciclovir treatment. The arrows indicate the day of treatments with NTS-polyplex nanoparticles carrying the plasmid pORF-HSVtk. GCV (70 mg/kg of body weight, i. p.) was administered daily. (**A, B, C**) Graphs of tumor-growing rate and tumor weight at the end of the study. (**D**) Photographs showing the dissected tumors from animals without treatment (control) or treated with local (L) or intravenous (IV) injections of NTS-polyplex nanoparticles harboring the plasmid pORF-HSVtk. (**E**) Representative agarose gel showing the presence of amplicons for HSVtk and 18S after a RT-PCR assay. *n = 5* independent experiments. *P<0.05 when compared with untransfected and GCV untreated cells. The difference in tumor growths was analyzed using a two-way ANOVA, and the difference in tumor weights was analyzed using a one-way ANOVA and *post-hoc* Bonferroni test.

Xenografts of 3×10^6^ of MDA-MB-231 cells in the subcutaneous layer of athymic mice spontaneously evolved to form tumors; once tumors measured 100 mm^3^, we injected NTS-polyplex nanoparticles harboring the plasmid pEGFP-N1 or pORF-HSVtk into the tumors or via the bloodstream every two days. Tumors in animals that were injected with NTS-polyplex nanoparticles carrying the plasmid pEGFP-N1 grew similar to untreated tumors (Figure 5A and 5B). In contrast, the local or intravenous treatment with NTS-polyplex harboring the plasmid pORF-HSVtk and the daily GCV injection (70 mg/kg of body weight; i.p.) significantly inhibited the growth of the tumors (Figure 5A and 5B). The differences were significantly different from day 3 (local treatment) and from day 5 (intravenous treatment) when compared with the control groups. At the end of the experiment, the tumor size was significantly reduced (*P*<0.001) by either the local transfection (60±8%) or the intravenous transfection (55±28%) when compared with the untreated and GFP controls. The weight of the tumors in the animals with local (0.28±0.07 g) or intravenous (0.23±0.05 g) treatment significantly decreased (*P*<0.05) by 55% compared with the controls (0.63±0.04 g) at the end of the study (Figure 5C–D). At this time, RT-PCR analysis showed HSVtk gene expression in tumors locally or intravenously treated with NTS-polyplex harboring the plasmid pORF-HSVtk and GCV injections (Figure 5E). This result suggests that the therapeutic effect was caused by the activation of the HSVtk suicide system.

### Targeted HSVtk Gene Delivery by NTS-polyplex Nanoparticles and Ganciclovir Treatment Induce Apoptosis in MDA-MB-231 Cell Tumors in Athymic Mice

**Figure 6:**
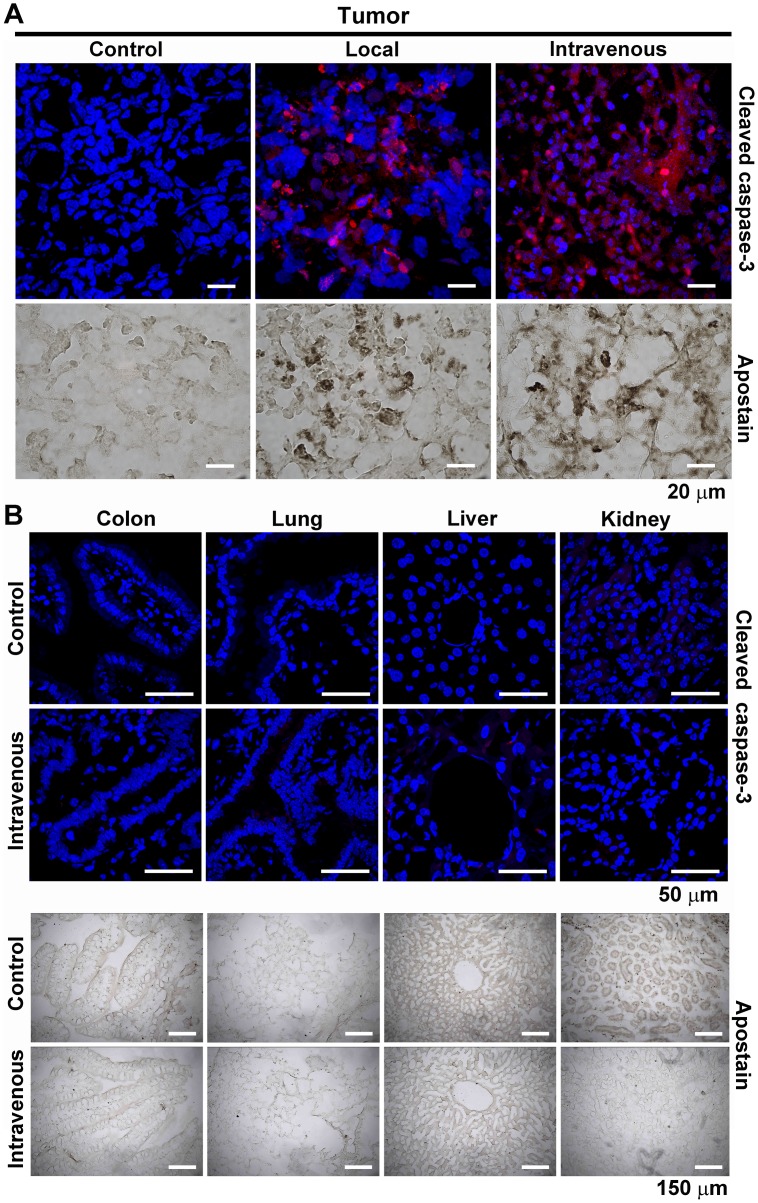
Presence of apoptosis in MDA-MB-231 cell tumors after HSVtk transfection and ganciclovir treatment. NTS-polyplex nanoparticles carrying the plasmid pORF-HSVtk were administrated locally or intravenously every two days. GCV (70 mg/kg of body weight, i. p.) was administered daily. The representative micrographs show the immunofluorescence against cleaved caspase-3 (red fluorescence) in cells that were counterstained with Hoechst 33258 (blue fluorescence). Representative bright field micrographs of condensed chromatin of apoptotic bodies were stained with Apostain.

The presence of cleaved caspase-3 immunoreactivity and condensed chromatin in tumors showed apoptosis activation by the treatment with NTS-polyplex harboring the plasmid pORF-HSVtk and GCV injections (Figure 6A). In contrast, none of these markers were found in tissues with the constitutive expression of NTSR1 (colon) or in organs that were involved in clearance (liver and kidneys) or lungs, in mice that were intravenously treated with NTS-polyplex-HSVtk and GCV (Figure 6B). These results show that the apoptotic effect that was caused by the intravenous treatment with NTS-polyplex nanoparticles harboring the plasmid pORF-HSVtk and GCV is preferentially exerted on tumors, as we previously demonstrated in a murine neuroblastoma model [24].

## Discussion

Our results demonstrate for the first time that the targeted HSVtk gene delivery by NTS-polyplex nanoparticles through the bloodstream inhibits the growth of tumors in a TNBC animal model with human MDA-MB-231 cells. Increased cleaved caspase-3 immunoreactivity and Apostain that is associated with HSVtk mRNA expression in transfected tumors indicate that apoptosis was involved in the tumoricidal effect of the HSVtk-GCV system, which was supported by our *in vitro* results and previous results [24], [41]. Although distant metastases (lung, bone, brain, liver) are most common in TNBC, cutaneous metastases have also been reported [50], [51]. For this reason, as well as to facilitate the experimental approach, we performed the xenograft of MDA-MB-231 cells in the subcutaneous tissue. Importantly, tumor-targeted NTS-polyplex nanoparticles were able to reach this metastatic rare site and produce the therapeutic effect in agreement with a previous work [24]. The presence of functional NTSR1 in MDA-MB-231 cells contributed to the transfection of the plasmids pORF-HSVtk and pEGFP-N1. This contention is supported by our internalization assays *in vitro*, which used competitive drugs for NTSR1 and an endocytosis blocker, and is consistent with previous findings *in vitro* and in animals [25], [28], [29].

We did not find evidence of apoptosis activation in peripheral organs that constitutively expressed (colon) or did not express (liver, lungs and kidneys) NTSR1 [23] after the intravenous administration of NTS-polyplex nanoparticles harboring the suicide HSVtk gene and GCV treatment. This result confirms previous findings in a neuroblastoma mouse model [24]. Previous studies have shown luciferase or GFP gene expression in colon cells after the intravenous injection of NTS-polyplex carrying those reporter genes [24], [32]. However, the gene expression in colon cells was significantly lower than that in the tumors, possibly caused by the difference in the number of NTSR1-bearing cells [24]. From the weak transgene expression in the colon, no apoptosis was observed in this organ after the intravenous transfection of the HSVtk gene and GCV treatment. The absence of apoptosis in the liver, lungs and kidneys is consistent with previous findings in a neuroblastoma mouse model [24] and can be explained by the inability of NTS-polyplex nanoparticles to transfect tissues lacking NTSR1 [24], [25], [28], [29]. Despite the absence of transfection, a recent study has shown accumulation of propidium iodide-labeled NTS-polyplex nanoparticles in the liver, spleen, lungs and kidneys 6 h after injection and their disappearance after 48 h [32]. The liver and spleen are the main organs with mononuclear phagocyte system that participate in the clearance of other polyplex nanoparticle systems by phagocytic uptake and filtration [52]–[54]. These evidences suggest that NTS-polyplex nanoparticles are taken up by cells mononuclear phagocyte system for degradation and clearance, but not necessarily for transfection [32]. These results support the safety of the HSVtk-GCV system in animals [24] and suggest that NTS-polyplex nanoparticles enable the gene transfection of NTSR1-expressing cells without causing undesirable side effects. This suggestion is supported by the absence of acute systemic inflammatory response or hepatic cytotoxicity after the intravenous injection of NTS-polyplex nanoparticles harboring the plasmid pEGFP-N1 [32]. Nevertheless, the immunogenicity of NTS-polyplex nanoparticles harboring a suicide gene should be further evaluated in cancer animal models using immunocompetent animals. Regardless of the outcome, NTSR1-bearing cells of healthy peripheral tissues can be protected from the deleterious effects of transgene expression using a cell-specific promoter or a tumor-selective promoter, such as the Mucin-1 promoter, to confine the therapeutic effect within the tumorous cells [55].

Our results that associate HSVtk expression with the death of MDA-MB-231 cells *in vitro* and *in vivo* suggest that activated GCV enables the therapeutic effect. The participation of the “bystander effect” is suggested by the finding that the therapeutic effect of HSVtk gene expression and GCV treatment on cell viability (49%±10%) was greater than the transfection efficiency (18.3±0.5%) as determined by flow cytometry using a reporter gene *in vitro* (Figure S1). Even though only a fraction of the cancer cells expresses the HSVtk gene, the expansion of the cell death can be achieved by the “bystander effect” [58]. This effect includes the transfer of toxic metabolic products that are derived from GCV through gap junctions, which are dependent on connexin expression [40], [59], [60]; however, MDA-MB-231 cells do not express connexins 43 and 26 [56], [57]. Alternatively, the “bystander effect” also includes mechanisms that are independent on connexins, such as endocytosis of apoptotic vesicles [61], release of soluble factors [62], [63], and stimulation of the immune system *in vivo* even in athymic mice due to the presence of B lymphocytes, macrophages and natural killer cells in them [64], [65]. These mechanisms might account for the inhibition (55–60%) of the tumor growth.

Two recent molecular targeted approaches have shown the same effectiveness as the tumor-targeted NTS-polyplex nanoparticles in TNBC cellular and xenograft models. The systemic delivery of liposomal short-chain ceramide that targets NTSR1 limits solid tumor growth in syngeneic and athymic murine models of breast adenocarcinoma [66] by inhibiting NTSR1 translocation into membrane microdomains, which ultimately inhibits the mitogen-activated protein kinase pathway [20]. Another strategy uses a mixture of antibodies that target EGFR (epidermal growth factor receptor), which are expressed in a subset of metaplastic breast cancer [67], to promote the lysosomal degradation of EGFR [68]. As a result, mAb mixtures inhibit the motility of TNBC cells, and cells arrest at G_1_, which can account for tumor inhibition [68]. Although the three molecular targeted strategies, including tumor-targeted NTS-polyplex nanoparticles, are independently effective, a combinatorial approach might be more efficient to limit fully or regress TNBC. In summary, tumor-targeted NTS-polyplex nanoparticles respond to the imperative demand to develop new molecular targeted strategies to control TNBC.

## Supporting Information

Figure S1 **Flow cytometry analysis of GFP expression in MDA-MB-231 cells with NTS-polyplex harboring the plasmid pEGFP-N1.** Representative dot plots of cells that were exposed 48 h to either the plasmid DNA-karyophilic peptide complex, a negative control (A), or the NTS-polyplex nanoparticles harboring the plasmid pEGFP-N1 (B). FSC-height = forward scatter and FL1-height = relative fluorescence intensity. The values at the bottom in each graphic correspond to the percentage of cells showing green fluorescence expression after excitation at 488 nm with a FACSCalibur flow cytometer (BD Biosciences; San Jose, CA, USA). The shown values are the mean ± SEM from 3 independent experiments.
(TIF)
