# Supplementary figures and images for: Suicide HSVtk Gene Delivery by Neurotensin-Polyplex Nanoparticles via the Bloodstream and GCV Treatment Specifically Inhibit the Growth of Human MDA-MB-231 Triple Negative Breast Cancer Tumors Xenografted in Athymic Mice
Source: PLoS One. 2014 May 13;9(5):e97151. doi: 10.1371/journal.pone.0097151 (PMC4019532; doi:10.1371/journal.pone.0097151)

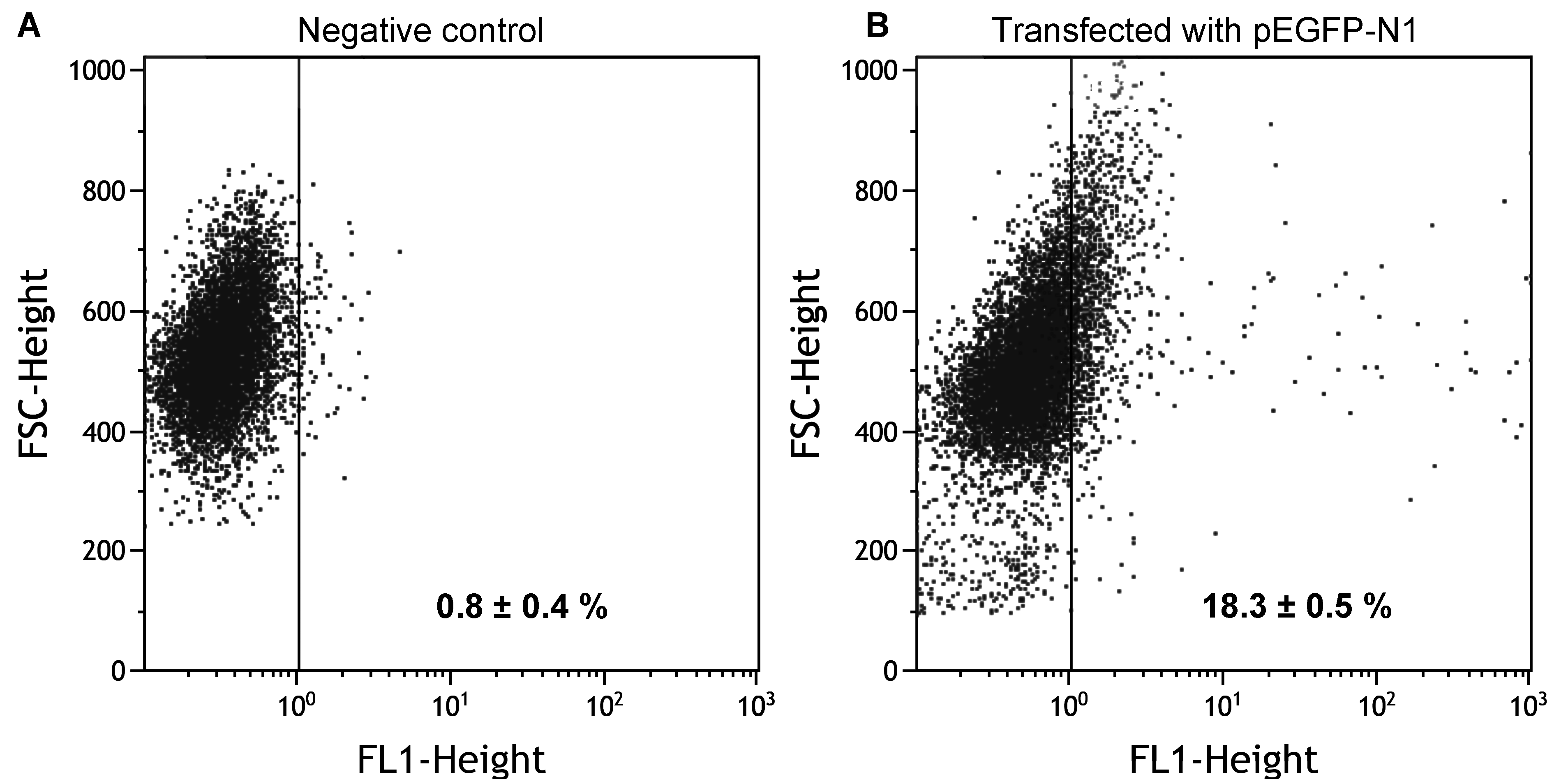

Supplement: Figure S1 — Flow cytometry analysis of GFP expression in MDA-MB-231 cells with NTS-polyplex harboring the plasmid pEGFP-N1. Representative dot plots of cells that were exposed 48 h to either the plasmid DNA-karyophilic peptide complex, a negative control (A), or the NTS-polyplex nanoparticles harboring the plasmid pEGFP-N1 (B). FSC-height = forward scatter and FL1-height = relative fluorescence intensity. The values at the bottom in each graphic correspond to the percentage of cells showing green fluorescence expression after excitation at 488 nm with a FACSCalibur flow cytometer (BD Biosciences; San Jose, CA, USA). The shown values are the mean ± SEM from 3 independent experiments. (TIF) [file pone.0097151.s001.tif]
